# Mental Health Literacy and Mental Health Information-Seeking Behavior in Iranian University Students

**DOI:** 10.3389/fpsyt.2022.893534

**Published:** 2022-06-13

**Authors:** Seyed Mohammad Hossein Mahmoodi, Masoud Ahmadzad-Asl, Mohammad Eslami, Mohadeseh Abdi, Yasamin Hosseini Kahnamoui, Maryam Rasoulian

**Affiliations:** ^1^Mental Health Research Center, School of Behavioral Sciences and Mental Health, Iran University of Medical Sciences, Tehran, Iran; ^2^Faculty of Medicine, Iran University of Medical Sciences, Tehran, Iran

**Keywords:** health literacy, information-seeking behavior, mental health literacy, trust, student

## Abstract

**Background:**

Inadequate Mental health literacy (MHL) is a problem worldwide. Research is limited in developing countries and about positive MHL. This study measured the disease-oriented MHL and positive MHL and investigated their association. In addition, this study explored the mental health information-seeking behavior among undergraduate students in Iran.

**Methods:**

This study recruited undergraduate students of Tehran public universities through multistage stratified random sampling to undertake an analytical cross-sectional study. MHL was measured using Mental Health Literacy Scale (MHLS) and Mental Health Positive Knowledge (MHPK). Participants' most used mental health information sources and their trust in them were also inspected.

**Results:**

A total of 543 students participated in this study. On average, the participants achieved about 65% of the total possible MHLS score, and 71% of participants showed sufficient MHPK score. The “internet” was the most used source for receiving and searching for mental health information. The most trusted source was “health care staff”. This study detected no correlation between disease-oriented MHL and positive MHL.

**Conclusions:**

Mental health literacy of Iranian students still seems to be insufficient. As disease-oriented MHL and positive MHL were not correlated, specific educational interventions for each domain are needed. Although the internet is the main source of mental health knowledge, the trust of student in it is low. This issue should be taken into consideration in designing online educational interventions.

## Introduction

Mental health literacy (MHL) is receiving increasing attention as a modifiable contributing factor in mental health. Jorm et al. coined the term mental health literacy (MHL) in 1997, inspired by the well-known concept of health literacy (1, 2). Further investigators tried to expand the concept, and recently four domains are suggested to construct the MHL: (1) understanding good mental health, i.e., mental health promotion, which is also known as positive MHL; (2) being familiar with mental disorders and treatments; (3) stigma against mental illness and treatment; and (4) help-seeking behavior competency (3).

Low MHL is related to various adverse mental health outcomes and is considered a significant contributing factor in alleviating mental problems and improving the mental health of individuals and the public (1, 4–7). Inadequate MHL, however, is a global problem, especially in developing countries (4, 6). Studies in the Middle East indicate poor MHL even in health care staff (4, 8–11). Similarly, the limited investigations in Iran showed low depression literacy and high stigma among medical students and other individuals (12–16). Most of the MHL studies around the world are disease-oriented, that is, they are focused on mental disorders, stigma, and help-seeking (3, 6). Positive MHL, therefore, is rarely studied, and it has not been evaluated in Iran.

Mental health information-seeking behavior includes the main channels people use for searching or receiving mental health information and how they assess and trust them (17). In Iran, there is an evident increase in using the internet as a source of health information (12, 18, 19). Iran's internet infrastructure has developed considerably in recent years; hence an updated evaluation of mental health information-seeking behavior in adults is needed.

Despite the number of MHL research, most studies have been conducted in Western countries and are limited to the awareness of depression and schizophrenia (4, 6). Many studies have used vignette-based instrument which is argued as weak methodology (1, 4). In the available studies on MHL, positive MHL is a neglected dimension. Additionally, the relationship between disease-oriented MHL and positive MHL remains unknown. Another gap is that the best methods to educate MHL in Iranians are not identified despite the use of several evidence-based interventions in developed countries (20–22). Understanding students' mental health information-seeking behavior is necessary to design more efficient educational programs, but it is less inspected, especially in developing countries.

This study aimed to (1) assess the level of disease-oriented MHL and positive MHL and examine their correlation in an Iranian population, and (2) explore participants' mental health information-seeking behavior.

## Methods

### Study Design and Population

This cross-sectional research is carried out in Tehran, the capital of Iran, from April to December 2019. Undergraduate students of public universities in Tehran were the study's target population. All students with basic internet skills could be included. There were no exclusion criteria.

### Sampling

Undergraduate students of Tehran public universities were recruited by multistage random sampling. Stratums were the six major disciplines that students can apply for at the beginning of their university education which are as follows: Mathematics & Physics, Natural Sciences, Humanities, Arts, Foreign Languages, and Career & Technical Education. The sample size of each discipline was calculated based on the number of admitted students from each discipline in the last 4 years which was extracted from annual application forms. One or two universities were selected randomly as clusters from each stratum. Likewise, at each university, some classes were chosen randomly as clusters from the table of the semester's active lessons. The whole of each selected class was included in the study. A sample size of 475 students was calculated using the formula of estimating a mean.

### Assessment

An online form was prepared for data collection. It contained an informed consent form, background data checklist, mental health information-seeking behavior questions, and two MHL questionnaires. The checklist assessed respondents' history of mental health services use or acquaintances with mental illness. Respondents were also asked to specify whether they were originally from a village, a small town, or a big city and indicate their parents' education level. We also asked them to specify the number of their roommates and rooms at their homes to calculate the household crowding index, which is used as an indirect indicator of participants' socioeconomic status. This continuous variable can be divided into three categories <1, 1–2, and more than 2. Higher numbers and especially numbers above 2 are correlated to lower socioeconomic and worse health status (23). The design of health information-seeking behavior questions was inspired by some questions of the Health Information National Trends Survey and assessed information sources that participants mainly used to passively receive and actively search for mental health topics (24). Participants were also requested to indicate how often they can trust each source on Likert's 5 points scale.

### Measurements

*The Mental Health Literacy Scale* (MHLS), introduced by O'Connor and Casey in 2015, assesses 3 of 4 dimensions of MHL, including knowledge about mental disorders, stigma, and help-seeking behavior (25). It is reported as both a valid and reliable tool with Cronbach's alfa of 0.873 and test-retest reliability (*r* = 0.797, *p* < 0.001). The original version has 35 questions with a 4 or 5 points Likert scale. The Persian version includes 23 questions and a possible score of 23 to 106, and is reported as a valid and reliable version (26). According to a systematic review that evaluated various MHL measures in 2016, MHLS showed strong properties and was one of the recommended instruments (20).

*Mental Health Positive Knowledge* (MHPK) is invented in 2017 and is the first instrument to evaluate positive MHL, i.e., knowledge about good mental health, which is one of the four dimensions of MHL. The average of all ten short questions of this instrument makes up the final score from 0 to 5. As a preliminary cut-off, developers considered lower than 4.00 averages as an index of insufficient positive MHL. They showed its validity and reliability with McDonald's omega of 0.84. The Persian version is also valid and reliable, with Cronbach's alfa of 0.81 (27).

### Statistical Analysis

Mean and the standard deviation is used to describe MHL levels. The association between MHLS and MHPK was tested by using Pearson's correlation. The statistical significance and power level were considered 0.05 and 80%, respectively. SPSS Statistics for Windows, version 16 (SPSS Inc., Chicago, Ill., USA) was used.

## Results

A total of 543 undergraduate students participated in this study; background characteristics are presented in Table 1. The response rate was 30%.

**Table 1:** Background characteristics and associated frequencies (%); for age and household crowding index the mean (±SD) is presented.

Age	20.75 (±2.74)
Household crowding index	1.25 (±0.62)
**Gender**
Females	327 (60.3%)
Males	215 (39.7%)
**Mother's university education**
Yes	223 (41.1%)
No	319 (58.9%)
**Father's university education**
Yes	264 (49.0%)
No	274 (51.0%)
**Habitat of origin**
Village	17 (3.2%)
Town	132 (24.5%)
City	390 (72.3%)
**Mental illness in acquaintances**
Yes	160 (29.7%)
No	379 (70.3%)
**Mental health service use**
No	350 (64.7%)
Yes	191 (35.3%)

*SD, standard deviation*.

### Mental Health Literacy

The mean (±SD) MHLS and MHPK were 69.59 (±7.77) and 4.09 (±0.73), respectively. There was no statistically significant difference in MHLS scores between study branches (*p* = 0.054), but MHPK was significantly lower in the “Career & Technical Education” branch (*p* = 0.004; Table 2). According to the preliminary cut-off, the frequency of students who had an insufficient level of MHPK was 151 (29.2%).

**Table 2:** The frequency of undergraduate students in each study branch and their mental health literacy scores.

		**MHLS**	**MHPK**
	**Frequency**	**(mean ±SD)**	**(mean ±SD)**
Mathematics & physics	133	70.48 ± 7.85	4.12 ± 0.66
Natural sciences	129	70.02 ± 7.47	4.17 ± 0.69
Humanities	117	70.13 ± 7.43	4.23 ± 0.54
Arts	24	70.56 ± 8.63	3.92 ± 0.88
Foreign languages	13	65.76 ± 5.76	4.00 ± 1.37
Career & technical	124	67.98 ± 8.10	3.88 ± 0.84
education
Total	543	69.59 ± 7.77	4.09 ± 0.73

*MHLS, Mental Health Literacy Scale; MHPK, Mental Health Positive Knowledge; SD, standard deviation*.

Parents' higher education showed a significant association with higher MHLS (*p*-values <0.001) but not MHPK. A mental illness in acquaintances was also associated only with higher MHLS (*p*-value < 0.001). In contrast, females scored significantly higher on MHPK (*p*-value < 0.001) but not MHLS. History of mental health service use showed significant associations with better scores in both measures (both *p*-values < 0.001). Finally, no significant correlation was detected between MHPK and MHLS scores.

### Mental Health Information-Seeking Behavior

In receiving mental health information, the most used mediums were “internet-based social media” and “school or university education”. The most used sources to search for mental health information were “search engines and websites” and “health care staff”. Students who preferred “School or University Education” or “health care staff” to find information showed the highest MHLS, and those whose choice was “internet-based social media” or “traditional or herbal healer” scored lowest (*p* = 0.002). The most trusted source was “health care staff” followed by “books, magazines or newspapers”. On the other hand, “television & radio”, “internet-based social media”, and “other smartphone applications” were among the least trusted mediums (Table 3).

**Table 3:** The percentage of using each source as the main source for receiving and searching for mental health information and rating of trust from 1 (lowest) to 5 (highest) to each one.

	**Main**	**Main**
**Sources of mental**	**receiving**	**searching**	**Trust**
**health information**	**source**	**source**	**(mean ±SD)**
School or University	16.7%	3.0%	3.27 ± 0.97
Education
Books, magazines	12.2%	8.0%	3.51 ± 0.81
or newspapers
Family or friends	11.9%	8.3%	3.04 ± 0.87
Traditional or	2.0%	3.5%	2.92 ± 1.07
herbal healer
Health care staff	7.2%	12.8%	4.00 ± 0.83
Television or radio	8.7%	0.6%	2.98 ± 0.94
Search engines	16.1%	53.1%	3.35 ± 0.72
& websites
Internet-based	24.3%	10.0%	2.78 ± 0.90
social media
Other smartphone	0.9%	0.7%	2.36 ± 0.85
applications

*SD, standard deviation*.

## Discussion

On average, participants achieved about 65% of the total possible disease-oriented MHL score. According to the developers' preliminary cut-off, about 30% of participants showed insufficient positive MHL (7). The most used sources for receiving and searching for mental health information were “Internet-based Social Media” and “Search engines and websites”, respectively. The most trusted source was “health care staff”. No correlation was detected between disease-oriented MHL and positive MHL.

Although there are no reference values for MHL in Iranian populations, its level in this study seems to be unsatisfactory, regarding that respondents have not achieved around 35% of the total MHLS score. In a previous investigation using the Persian version of MHLS, the results were lower compared to the current study (26). As participants in our study were young and educated, such a difference is expected. Various studies have concluded that the MHL of different Iranian populations is not adequate (14–16, 28). For example, the depression literacy of Tehran medical students is described as very low in 2015 and of Tehran residents as low in 2019 (12, 13). These results are consistent with known unsatisfactory MHL in the middle east and developing countries (4, 8, 10, 11).

Females have shown higher MHL than males in most of the investigations; however, this result is not replicated in the educated sample of this study and the 2015 study of Tehran medical students (4, 6, 12, 13). To explain the similar MHL level in male and female students in these two studies it can be hypothesized that university education may improve males' MHL and therefore eliminates such gender differences. It would be consistent with the known improving effect of education on MHL (6). Furthermore, in the current study, “school and university education” was one of the most used and trusted sources of mental health information.

Similar to previous findings, in this study, the lower socioeconomic status estimated by the household crowding index was correlated with lower MHLS, although its strength was minimal (4). According to the literature, rural populations tend to show lower MHL (4). In this study, participants from villages showed less but not statistically significant average MHLS scores. There is no MHL investigation in the rural populations of Iran.

Measuring positive MHL is necessary to attain a comprehensive picture of individuals' MHL but hasn't been studied until recently (3, 6, 7). Our educated participants showed lower positive MHL compared to the previous study on Norwegian youth (7). Such differences may arise simply from instrument translation effects or may be considered as a replication of known differences between developing and developed countries' MHL. Similar to Norwegian youth, females showed higher positive MHL than males in this study.

Examining the association between disease-oriented MHL and positive MHL showed no correlation. It has implications for mental health education interventions in the way that positive MHL may not be improved through conventional disease-oriented mental health education and may need independent interventions.

Similar to developed and some developing countries, the internet was the most used source of mental health information in this study (19, 29, 30). However, television which was as important as the internet in previous surveys is rarely used now (12, 18). However, according to investigations in other countries, increasing the use of the internet for mental health information-seeking is not necessarily translated to more help-seeking behavior so it needs to be explored in Iran (31).

Although respondents showed shallow trust in “Internet-based Social Media”, they declared that they receive most of their mental health information from this channel. It suggests that users may be exposed to a massive amount of inaccurate data. It is known that incorrect health information is harmful, and it can be addressed by improving people's e-health literacy, another area of future research in Iranian populations (32).

Unlike social media, participants show high trust in health staff, but they don't receive much information from them. This can raise questions about the attitude and practice of staff in improving clients' health knowledge and clients' potential barriers to receiving such education. Sayarifard et al. in their 2015 study of Tehran medical students discussed that the low trust in the mental health system might be the cause of low help-seeking (12). However, the high trust in health care staff in the current study is contrary to that explanation and shows the need for further exploration.

This study was novel to evaluate the MHL of different study branches of university students, assess positive MHL, and examine its correlation to disease-oriented MHL. Using the random sampling method is a strength. A limitation of the findings is the considerable reduction of the number of questions in the Persian version of MHLS which has happened during its translation and validation process (26). Although the resulting version has satisfactory psychometric properties, its coverage has been reduced compared to the original version. Nevertheless, using MHLS which is not limited to one disorder like depression and is not vignette-based is a strength of the current study. Finally, our response rate is less than what is expected in academic samples and this is a potential source of selection bias (33).

This study provides a comprehensive evaluation of mental health literacy (MHL) in a sample of Iranian undergraduate university students and shows that some deficiencies are still noticeable. Current educational interventions, therefore, need to be improved. Regarding the significant portion of participants with insufficient positive MHL, it can also be considered to create educational programs focused on mental health improving factors. Our findings don't show a correlation between positive MHL and disease-oriented MHL so designing specific educational materials for each domain seems reasonable. Implementing such programs and evaluating their efficacy can be the next move in future research in this field. For designing such interventions, our results on mental health information-seeking behavior and trust would help to find the most efficient mediums.

## Data Availability Statement

The raw data supporting the conclusions of this article will be made available by the authors, without undue reservation.

## Ethics Statement

The studies involving human participants were reviewed and approved by the Ethics Committee of the Iran University of Medical Sciences with the reference number of IR.IUMS.FMD.REC.1397.173. The patients/participants provided their written informed consent to participate in this study.

## Author Contributions

SM contributed to the design of the work, data acquisition, analysis, interpretation, and drafting of the manuscript. MA-A and MR contributed to the conception and design, data interpretation, and revising of the manuscript critically. ME, MA, and YH contributed to designing the study, data collection, statistical analysis, and constructing the final manuscript. All authors approved the final version.

## Conflict of Interest

The authors declare that the research was conducted in the absence of any commercial or financial relationships that could be construed as a potential conflict of interest.

## Publisher's Note

All claims expressed in this article are solely those of the authors and do not necessarily represent those of their affiliated organizations, or those of the publisher, the editors and the reviewers. Any product that may be evaluated in this article, or claim that may be made by its manufacturer, is not guaranteed or endorsed by the publisher.
